# Large Inverted Duplications in the Human Genome Form via a Fold-Back Mechanism

**DOI:** 10.1371/journal.pgen.1004139

**Published:** 2014-01-30

**Authors:** Karen E. Hermetz, Scott Newman, Karen N. Conneely, Christa L. Martin, Blake C. Ballif, Lisa G. Shaffer, Jannine D. Cody, M. Katharine Rudd

**Affiliations:** 1 Department of Human Genetics, Emory University School of Medicine, Atlanta, Georgia, United States of America; 2 Department of Biostatistics and Bioinformatics, Emory University School of Public Health, Atlanta, Georgia, United States of America; 3 Signature Genomic Laboratories, PerkinElmer, Inc., Spokane, Washington, United States of America; 4 Department of Pediatrics, University of Texas Health Science Center at San Antonio, San Antonio, Texas, United States of America; 5 The Chromosome 18 Registry and Research Society, San Antonio, Texas, United States of America; Duke University, United States of America

## Abstract

Inverted duplications are a common type of copy number variation (CNV) in germline and somatic genomes. Large duplications that include many genes can lead to both neurodevelopmental phenotypes in children and gene amplifications in tumors. There are several models for inverted duplication formation, most of which include a dicentric chromosome intermediate followed by breakage-fusion-bridge (BFB) cycles, but the mechanisms that give rise to the inverted dicentric chromosome in most inverted duplications remain unknown. Here we have combined high-resolution array CGH, custom sequence capture, next-generation sequencing, and long-range PCR to analyze the breakpoints of 50 nonrecurrent inverted duplications in patients with intellectual disability, autism, and congenital anomalies. For half of the rearrangements in our study, we sequenced at least one breakpoint junction. Sequence analysis of breakpoint junctions reveals a normal-copy disomic spacer between inverted and non-inverted copies of the duplication. Further, short inverted sequences are present at the boundary of the disomic spacer and the inverted duplication. These data support a mechanism of inverted duplication formation whereby a chromosome with a double-strand break intrastrand pairs with itself to form a “fold-back” intermediate that, after DNA replication, produces a dicentric inverted chromosome with a disomic spacer corresponding to the site of the fold-back loop. This process can lead to inverted duplications adjacent to terminal deletions, inverted duplications juxtaposed to translocations, and inverted duplication ring chromosomes.

## Author Summary

Chromosomes with large inverted duplications and terminal deletions cause neurodevelopmental disorders in children. These chromosome rearrangements typically involve hundreds of genes, leading to significant changes in gene dosage. Though inverted duplications adjacent to terminal deletions are a relatively common type of chromosomal imbalance, the DNA repair mechanism responsible for their formation is not known. In this study, we analyze the genomic organization of the largest collection of human inverted duplications. We find a common inverted duplication structure, consistent with a model that requires DNA to fold back and form a dicentric chromosome intermediate. These data provide insight into the formation of nonrecurrent inverted duplications in the human genome.

## Introduction

Inverted duplications adjacent to terminal deletions are a relatively common copy number variation (CNV) first identified by chromosome banding [1]. With the rise in clinical array testing, such rearrangements are now recognized more often by the characteristic copy number gain adjacent to a terminal loss detected via microarray [2], [3]. Inverted duplications adjacent to terminal deletions have been described on nearly every chromosome end and, depending on the genes involved, can lead to a range of clinical phenotypes, including developmental delay, intellectual disability, autism, and birth defects [2], [4], [5], [6], [7], [8]. Moreover, large inverted duplications are a source of oncogene amplification in cancer genomes [9], [10], [11], [12], [13]. Large inverted duplications adjacent to deletions are also present in bacteria, yeast, protozoa, and worm genomes [14], [15], [16], [17], [18], [19], [20], [21] and are therefore a major cause of genomic imbalance in many cell types.

Several models are proposed to explain the formation of inverted duplications adjacent to terminal deletions in the human genome, and most include a dicentric chromosome step, as first described by McClinock [22]. One mechanism relies on homologous recombination (HR) between segmental duplications and is based on the inverted duplication and terminal deletion of the short arm of human chromosome 8. This recurrent rearrangement is always maternal in origin and occurs when normal and inverted homologous chromosomes 8 recombine during meiosis I [23], [24]. Recombination between highly identical inverted segmental duplications on 8p produces a dicentric chromosome and an acentric fragment. The acentric fragment is usually lost, but the dicentric chromosome may be recovered after breakage between the two centromeres and addition of a new telomere. This results in a chromosome with a 7.0-Mb terminal deletion, 5.5-Mb intervening normal copy region, and a proximal inverted duplication that varies in size, depending on the location of the dicentric chromosome break.

The mechanisms responsible for other human inverted duplications have remained elusive for a number of reasons. First, most deletion and duplication breakpoints are not recurrent, so the local genomic architecture underlying double-strand breaks does not point to a common rearrangement mechanism. Second, most inverted duplications adjacent to terminal deletions are characterized by array comparative genome hybridization (CGH) and/or fluorescence *in situ* hybridization (FISH), without sequencing of breakpoint junctions [6], [7]. Thus, conclusions drawn from such examples are missing key data that could shed light on specific DNA repair processes. In those inverted duplication junctions that have been sequenced, there are no obvious segmental duplications to suggest non-allelic homologous recombination (NAHR) [4], [5]. Thus, some other mechanism likely explains these nonrecurrent chromosome rearrangements, which make up the largest fraction of human inverted duplications.

The timing of inverted duplication formation is another important open question when considering rearrangement mechanism. Most constitutional (non-tumor) inverted duplications are present in a non-mosaic state, consistent with an event that occurred during meiosis or mitosis of the early embryo [2], [4], [6], [7]. Rare mosaic inverted duplications support a mitotic origin for inverted duplication formation [25], [26], and models for both meiotic and mitotic processes have been proposed [6], [9]. Some of the most striking evidence for mitotic inverted duplication formation comes from copy number studies of human blastomeres. CNV analyses of single cells from the same embryo have revealed inverted duplication chromosomes and their reciprocal terminal deletion products, consistent with a mitotic embryonic origin for inverted duplications [27], [28].

In this study, we analyzed the largest cohort of naturally occurring human inverted duplications. We fine-mapped the breakpoints of 50 inverted duplications using custom high-resolution array CGH. In 25/50 of the chromosome rearrangements, we sequenced breakpoint junctions via long-range PCR, custom target capture, and next-generation sequencing. Together, these breakpoint data point to a fold-back model of inverted duplication formation.

## Results

### Inverted duplication cohort

**Figure 1:**
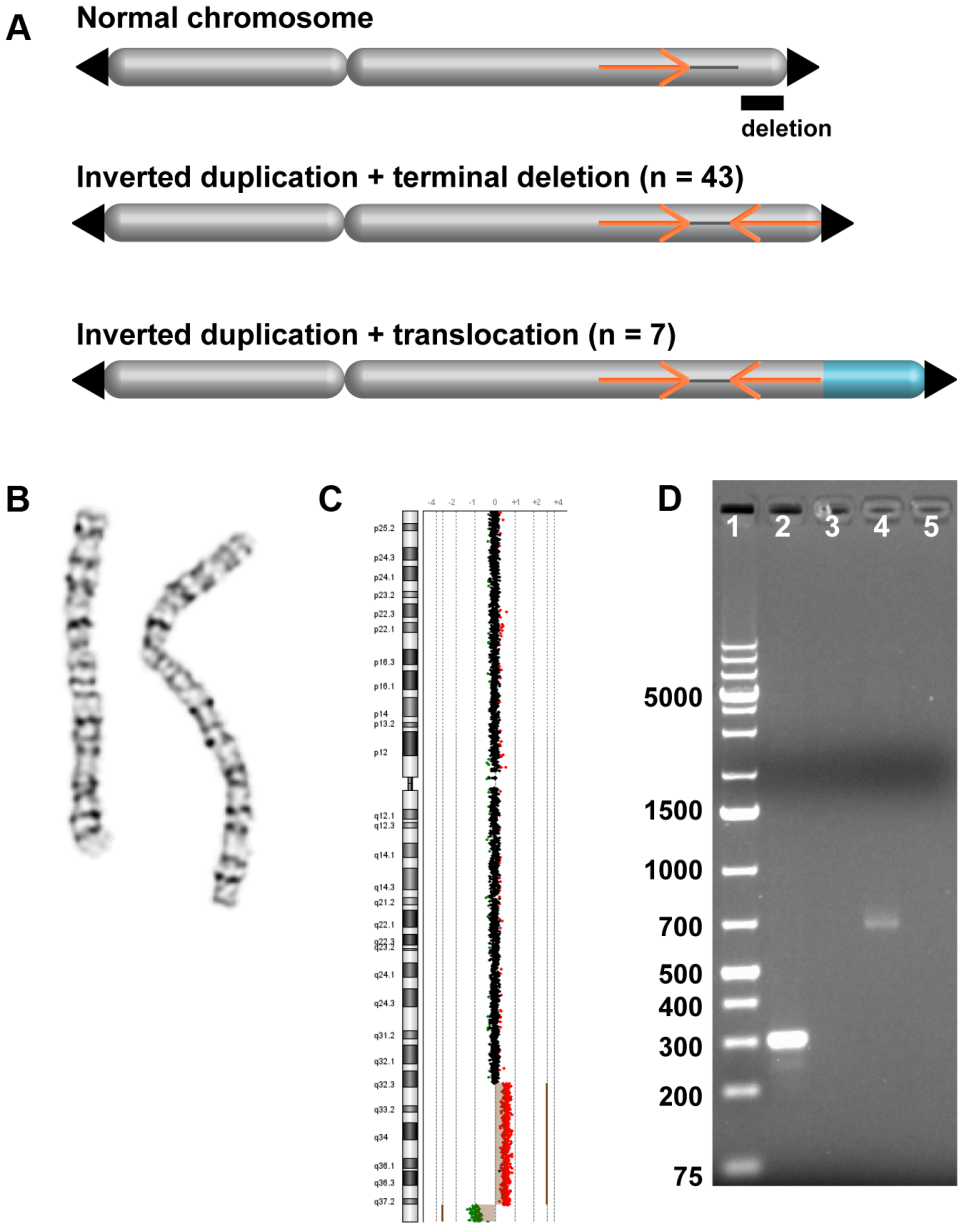
Inverted duplication organization. (A) Model of duplicated sequences (orange arrows) separated by disomic spacer sequence (grey line). The end of the inverted duplication may terminate in a telomere (black triangle) or a translocated chromosome (blue). The site of the terminal deletion is shown relative to a normal chromosome. (B) EGL044's inverted duplication of chromosome 2 is detectable by chromosome banding. (C) The 5.8-Mb terminal deletion and 42-Mb inverted duplication of chromosome 2 are detectable by low-resolution array CGH [53]. Note that the 2,047-bp spacer region is not visible. Log2 ratios of oligonucleotide probes are indicated by dots; normal-copy number (black), duplication (red), and deletion (green) regions are shown. (D) PCR of the disomy-inversion junction (lane 2) and the inversion-telomere junction (lane 4) amplifies genomic DNA from EGL044, but not control genomic DNA (lanes 3 and 5). Lane 1 is GeneRuler 1 kb Plus DNA ladder (Thermo Scientific Fermentas #SM1333).

**Figure 2:**
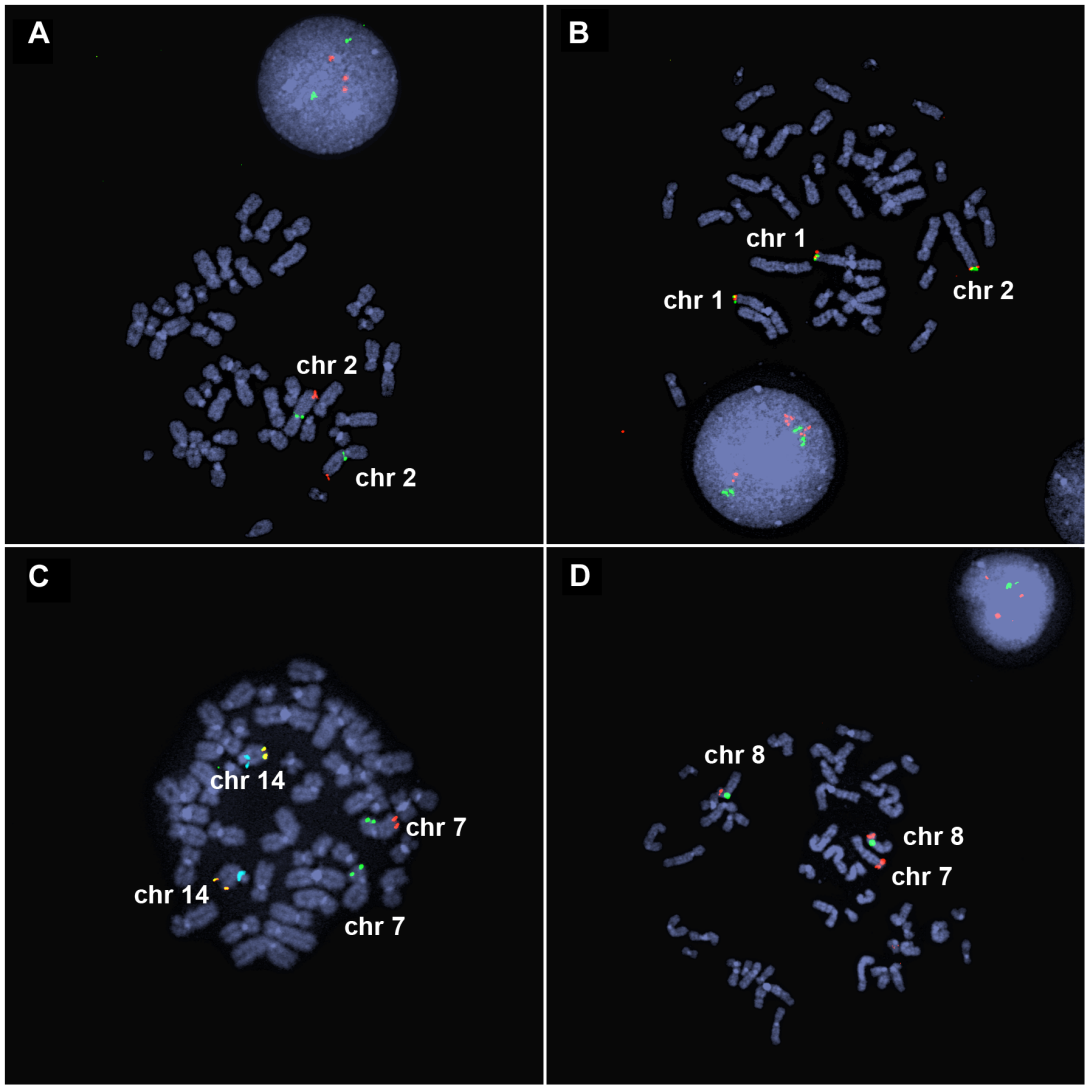
FISH analysis of inverted duplication translocation chromosomes. (A) EGL398's 3.1-Mb duplication of 2q37 is visible by interphase FISH. BAC probes RP11-206J15 (red) and RP11-1415N13 (green) hybridize to the duplicated and control regions on chromosome 2, respectively. Three red signals in the interphase nucleus indicate a duplication of chromosome 2q37. (B) BAC probes RP11-798H13 (red) and RP11-380E2 (green) hybridize to the ends of the normal chromosomes 1p and the end of the inverted duplication translocation chromosome in EGL398. (C) EGL399's terminal deletion of 7q is detected as loss of a red signal. Vysis ToTelVysion mix 7 (Abbott Molecular, #05J05-001) probes hybridize to the ends of chromosomes 7p (green), 7q (red), and 14q (yellow). The blue signals correspond to a control probe that hybridizes to chromosome 14q11. (D) BAC RP11-341D4 (red) hybridizes to the normal chromosomes 8p and the translocation of 8p on EGL399's inverted duplication translocation between chromosomes 7 and 8. The green signal corresponds to alpha satellite from the centromere of chromosome 8.

To capture a large collection of inverted duplications, we recruited 50 participants with pathogenic copy number variation (CNV) from Emory University, Signature Genomic Laboratories, and the Chromosome 18 Clinical Research Center. The children in our study carry nonrecurrent chromosome rearrangements that involve hundreds of genes per deletion or duplication, and they exhibit a range of phenotypes from developmental delay and intellectual disability to autism and other neurodevelopmental disorders. CNVs were initially detected via clinical cytogenetics testing, including array CGH, FISH, and/or chromosome banding (Figures 1 and 2). Individuals with inverted duplications adjacent to terminal deletions and their family members were referred to our study.

Forty-three subjects had a rearranged chromosome with a terminal loss and an adjacent gain, consistent with a simple inverted duplication adjacent to a terminal deletion. Seven had a terminal deletion adjacent to a duplication, plus a gain of another chromosome end, which when analyzed by FISH, turned out to be an unbalanced translocation juxtaposed to the inverted duplication (Figure 2). Parental samples were provided for 26/50 of the subjects in our study. Chromosome analysis and FISH revealed that 25/26 of inverted duplications were not present in a balanced or unbalanced form in either parent (Table S1). In one family (EGL396), the same inverted duplication was inherited from a similarly affected mother. Thus, most inverted duplications arise *de novo*.

The parental origin of the inverted duplication can shed light on the mechanism of chromosome rearrangement. To this end, we analyzed microsatellites in the deleted and duplicated regions from nine subjects and their parents (Table S2). In seven families there were sufficient informative markers to determine that the duplication and deletion were paternally inherited and that the duplication allele originated from the same chromosome as the deletion. For the families of 18q-119c and EGL106, only the mothers were genotyped. Microsatellites were consistent with a duplication of the paternal allele and retention of the maternal allele in the deleted region. These data support an intrachromosomal origin for inverted duplications that arose on the same allele as the original locus, rather than a duplication from the homologous chromosome.

### Breakpoint mapping and sequencing

**Figure 3:**
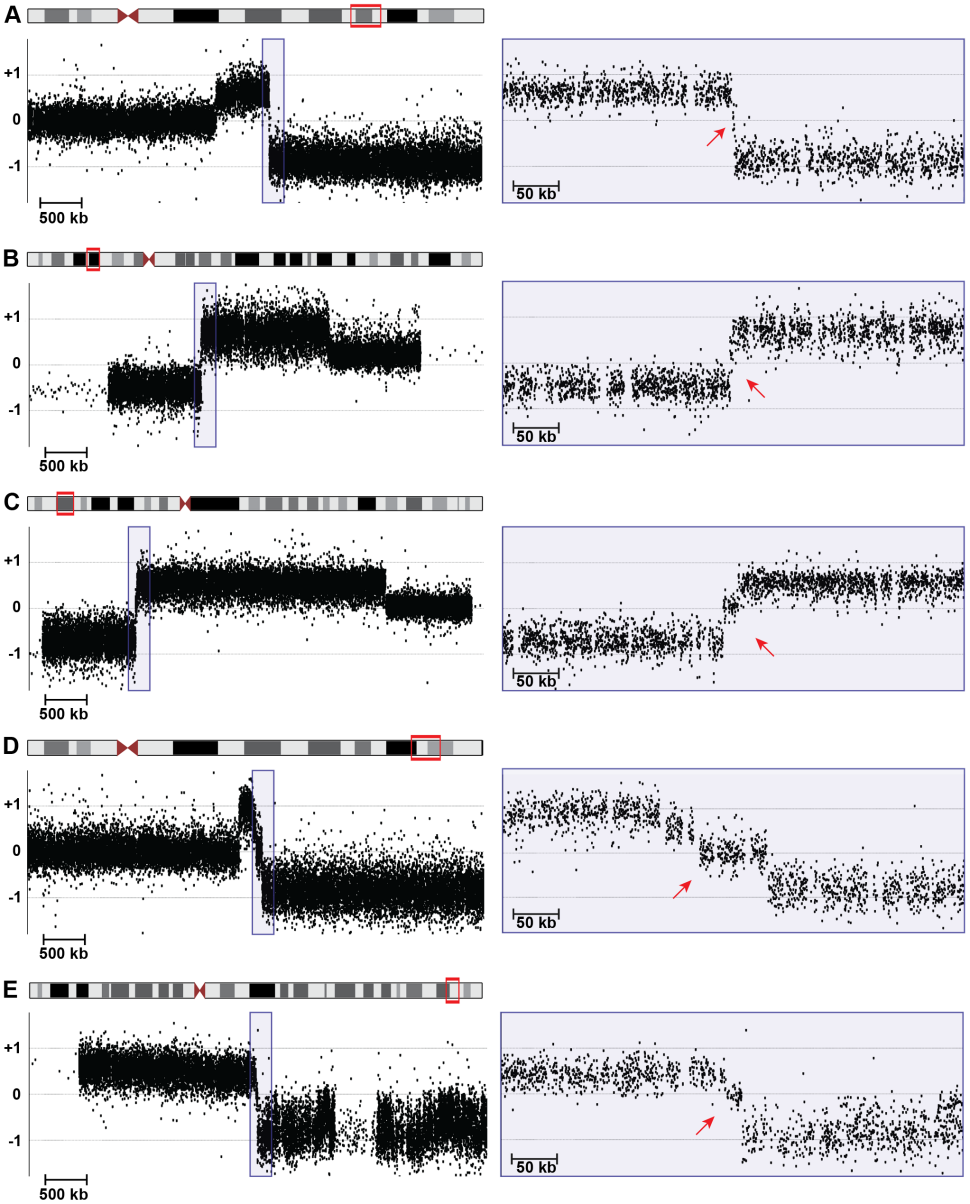
High-resolution array CGH identifies spacers. 5-Mb (left) and 400-kb (right) views of high-resolution array CGH data from (A) 18q-6c, (B) EGL106, (C) EGL104, (D) 18q-233c, and (E) SG_Tel_010 show 1,866-bp, 3,138-bp, 14,779-bp, 70,466-bp, and 14,779-bp spacers, respectively. Log2 ratios of probe signal intensity are shown as black dots. Boxed region on the left is expanded on the right. Red arrows point out disomic spacer regions between deleted and duplicated segments. Spacer sizes were determined by sequencing breakpoint junctions in (A)–(D), whereas the spacer in (E) was sized using breakpoints determined by array CGH only.

To refine deletion and duplication breakpoints, we fine-mapped CNVs with custom high-resolution microarrays (Figure 3). Oligonucleotide probes on the custom arrays are spaced one per ∼200 basepairs (bp), which in most cases resolve chromosome breakpoints to ∼1 kilobase (kb). However, repeat-rich regions and assembly gaps can limit array design, leading to poor probe coverage at some breakpoints. We identified deletion, duplication, and translocation breakpoints via array CGH as previously described [29]. Based on breakpoints predicted from our high-resolution array data, we designed long-range PCR, inverse PCR, SureSelect target enrichment, and next-generation sequencing experiments to sequence across breakpoint junctions (Table 1 and Table S1).

**Table 1:** Sequenced breakpoint junctions.

Subject	Chr	CNV type	Deletion	Duplication	Spacer	Capture method	Inheritance	Dis-inv junction	Inv-tel junction	Inv-tra junction
**SGTel022**	2q	Inv-dup term del	6,191,297	4,559,194	1,459	PCR	unknown	1		
**EGL044**	2q	Inv-dup term del	5,803,294	41,659,284	2,047	SureSelect and PCR	de novo	1	1	
**SGTel014**	2q	Inv-dup term del	3,284,385	8,846,045	9,316	PCR	de novo	1		
**EGL395**	2q	Inv-dup term del	3,262,416	5,006,517	2,914	PCR	de novo	1		
**EGL014**	4p	Inv-dup term del	561,480	2,462,234	3,428	PCR	unknown	1		
**SGTel013**	4p	Inv-dup term del	4,470,923	1,045,252	3,948	PCR	de novo	1		
**SGTel015**	5p	Inv-dup term del	18,804,928	27,181,320	3,993	PCR	unknown	1		
**EGL106**	5p	Inv-dup term del	25,750,936	1,405,992	3,138	PCR	unknown	1	1	
**EGL399**	7q	Inv-dup translocation	2,335,653	12,392	5,040	PCR	de novo			1
**EGL074**	9p	Inv-dup translocation	10,358,949	811,440	7,486	SureSelect and PCR	unknown	1		
**M397**	9p	Inv-dup translocation	10,481,181	1,461,954	3,450	SureSelect and PCR	unknown	1		1
**EGL104**	9p	Inv-dup term del	10,503,832	2,786,015	14,779	PCR	unknown	1	1	
**SGTel019**	13q	Inv-dup term del	1,496,671	6,628,755	9,009	PCR	unknown	1		
**18q-207c**	18q	Inv-dup term del	28,547,996	947,547	9,519	PCR	de novo	1	1	
**EGL099**	18q	Inv-dup term del	22,238,039	5,508,846	5,489	PCR	unknown	1		
**18q-26c**	18q	Inv-dup term del	20,952,219	211,862	766	SureSelect and Inverse PCR	de novo	1	1	
**18q-6c**	18q	Inv-dup term del	20,032,810	595,310	1,866	PCR	de novo	1	1	
**18q-34c**	18q	Inv-dup term del	20,009,964	8,936,902	4,035	PCR	de novo		1	
**18q-223c**	18q	Inv-dup term del	16,438,679	922,294	1,543	PCR	de novo	1	1	
**SGTel009**	18q	Inv-dup term del	14,869,902	4,787,035	700	PCR	unknown		1	
**18q-65c**	18q	Inv-dup term del	14,644,742	14,462,430	2,136	PCR	de novo	1	1	
**18q-139c**	18q	Inv-dup term del	12,951,972	1,899,319	951	PCR	de novo	1		
**18q-233c**	18q	Inv-dup term del	9,592,937	181,800	70,466	PCR	de novo	1		
**18q-107c**	18q	Inv-dup term del	8,539,434	5,703,158	1,822	PCR	de novo	1		
**M396**	18q	Inv-dup translocation	3,438,438	10,434	724	PCR	maternal			1

Sizes of deletions, duplications, and spacers in bp are shown. The numbers of sequenced disomy-inversion (Dis-inv), inversion-telomere (Inv-tel), and inversion-translocation (Inv-tra) junctions are listed. Spacers without a sequenced Dis-inv junction were measured from most distal duplicated probe to the most proximal deleted probe on the array. The full list of inverted duplication CNVs is provided in Table S1.

**Figure 4:**
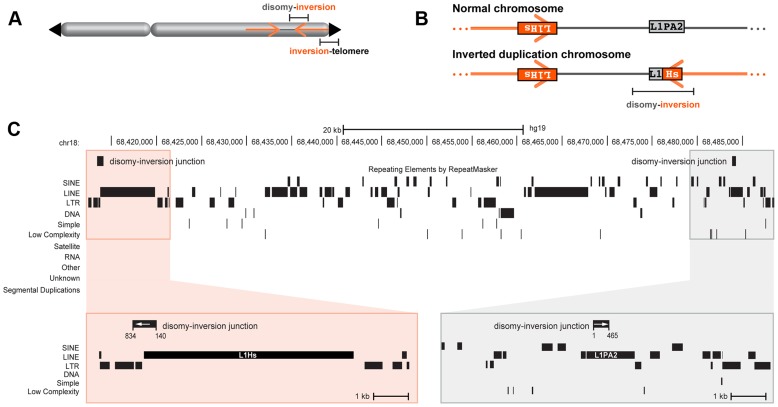
Inverted duplication junctions. (A) Location of disomy-inversion and inversion-telomere junctions in an inverted duplication terminal deletion chromosome. (B) 18q-233c's disomy-inversion junction spans a hybrid LINE made up of L1PA2 and L1Hs elements. On a normal chromosome 18, these elements are positioned in opposite orientation. (C) Local genomic context of 18q-233c's spacer and breakpoints relative to the reference genome assembly. The distal end of the disomic spacer (grey box) includes the L1PA2, and the proximal region corresponding to the beginning of the inverted duplication (orange box) includes the L1Hs. The disomy-inversion junction sequence (black rectangles with white arrows) aligns to the distal end of the spacer (positions 1–465 of the junction) and the start of the inverted duplication (positions 140–834 of the junction). Interspersed repeats are shown as black rectangles. No segmental duplications are present in the breakpoint regions.

Starting with breakpoints identified by high-resolution array, we designed PCR experiments to amplify 68 junctions [29], [30] (Figure 4 and Table S1). In some cases, there was not enough DNA to try multiple junction sequencing strategies. For other junctions that failed long-range and/or inverse PCR conditions, we performed targeted sequence capture with custom SureSelect libraries designed for our breakpoint regions of interest, followed by next-generation sequencing (see Methods). Of the 10 patient samples included in our SureSelect experiments, junctions from EGL044, EGL074, and M397 had sufficient paired-end and/or split read coverage to infer breakpoint structure, which we confirmed by Sanger sequencing.

Simple inverted duplications adjacent to terminal deletions have two breakpoint junctions: one from the non-inverted part of the chromosome to the start of the inverted duplication (disomy-inversion) and one from the end of the inverted duplication to the new telomere (inversion-telomere). Similarly, inverted duplications with unbalanced translocations have one disomy-inversion junction and one junction between the inverted duplication and the translocated chromosome (inversion-translocation). In both types of rearrangement, the terminal deletion corresponds to the region distal of the duplication (Figure 1). In total, we sequenced across 21 disomy-inversion junctions from 19 simple inverted duplications and two inverted duplications adjacent to translocations. We also sequenced 10 inversion-telomere junctions and three inversion-translocation junctions. All 34 of these sequenced junctions are present in the patient with the chromosome rearrangement, but not in a control genome, consistent with patient-specific junctions (Figure 1D). We aligned junction sequences to the human reference genome assembly to analyze the transitions across breakpoints and detect regions of microhomology and/or short inversions and insertions at junctions (Figure S1).

### Inverted duplication organization

**Figure 5:**
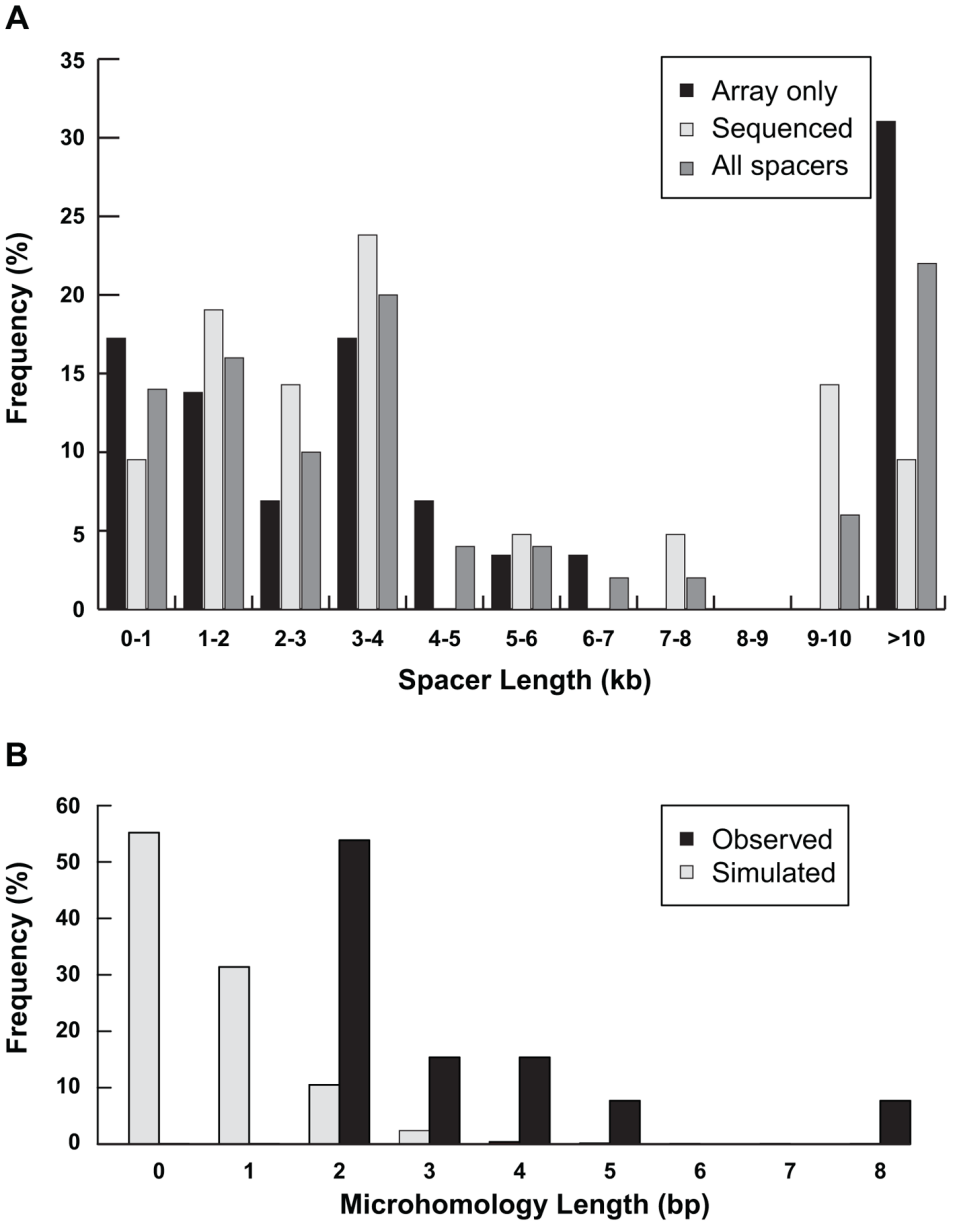
Characterization of spacers. (A) Distribution of lengths of spacers measured by high-resolution array CGH only (n = 29) or junction sequencing (n = 21) are plotted separately. The distribution of all 50 spacer lengths is also shown. (B) The amount of inverted microhomology observed at 13 sequenced disomy-inverted duplication junctions—2 bp (n = 7), 3 bp (n = 2), 4 bp (n = 2), 5 bp (n = 1), or 8 bp (n = 1)—are shown relative to the microhomology detected for 1,000 simulated spacers (see Methods).

Analysis of breakpoint junctions can point to mechanisms of chromosome rearrangement and modes of DNA repair. Remarkably, in all 21 sequenced disomy-inversion junctions, we found a short “spacer” region between the non-inverted and inverted segments (Figures 3 and 5). This region is 766–70,466 bp long (median = 3,428 bp) and is not duplicated; rather, it has a normal disomic copy number in the subject's genome. Since 20 out of 21 disomic spacers are less than 15 kb, it is not surprising that they were not detected by routine cytogenetics testing (Figure 1). Spacers that were not sequenced have a median size of 3,568 bp, as determined by array CGH (Table S1). Detection and analysis of spacers provide important clues to the mechanism of inverted duplications.

Previous studies from cancer genomes and model systems support a fold-back mechanism of duplication formation [9], [16], [17], [18], [20], [31]. In this scenario, an initial double-strand break (DSB) deletes the end of a chromosome, leaving an unprotected end without a telomere. DNA from this free end could resect, fold back on itself, and pair with a more proximal region of the chromosome, especially if the two regions share homologous sequence oriented in the reverse complement. If the fold-back mechanism is responsible for the inverted duplications in our study, we would expect to find direct sequence homology between the distal end of the disomic spacer and the start of the inverted duplication. When aligned to the normal reference genome, the breakpoint junction would share inverted homology between the distal end of the disomic spacer and the distal end of the region that is duplicated.

Analysis of the disomy-inversion junctions revealed sequence homologies between the end of the disomic spacer and the start of the inverted duplication. In three out of 21 sequenced junctions, homologous LINE or SINE repeats are present at the edges of the disomic spacer and the inverted duplication (Figure S2). Analysis of EGL104's disomy-inversion junction revealed 296 bp of sequence homology between 90% identical *Alu*Y elements that lie in opposite orientation as positioned in the reference genome. SGTel014's junction crosses an *Alu*Sx1 at the end of the disomic spacer to an *Alu*Sq2 in the duplicated segment; the *Alu*s are 82% identical over 296 bp. LINE elements flank 18q-233c's disomy-inversion junction in which a L1PA2 element at the end of the disomic spacer transitions to a L1Hs that is 95% identical across 330 bp at the junction (Figure 4). In all three of these rearrangements, the disomy-inversion transition occurs at homologous sites within the repetitive element, creating a hybrid repeat in the same orientation at the breakpoint junction.

Shorter inverted microhomologies are present in 13 of the remaining 18 disomy-inverted duplication junctions (Figure S1). The other five disomy-inversion junctions contain sequence insertions at the breakpoints. To determine whether the amount of inverted microhomology is greater than expected by chance, we simulated 1,000 spacers in the human genome. We counted the number of bp shared between the distal end of the spacer and the reverse complement of the other end of the sequence, representing the start of the inverted duplication (see Methods). Simulated spacers have 0–4 bp of microhomology, with no microhomology at 55% of all simulated junctions. On the other hand, the 13 sequenced disomy-inversion junctions had 2–8 bp of inverted microhomology (Figure 5B). Microhomology of greater than or equal to 2 bp is enriched at sequenced spacer junctions compared to simulated junctions (p = 2.7×10^−12^). Together, these data suggest that short inverted sequences at disomy-inversion junctions are an important feature of human inverted duplications.

### Complex rearrangements

**Figure 6:**
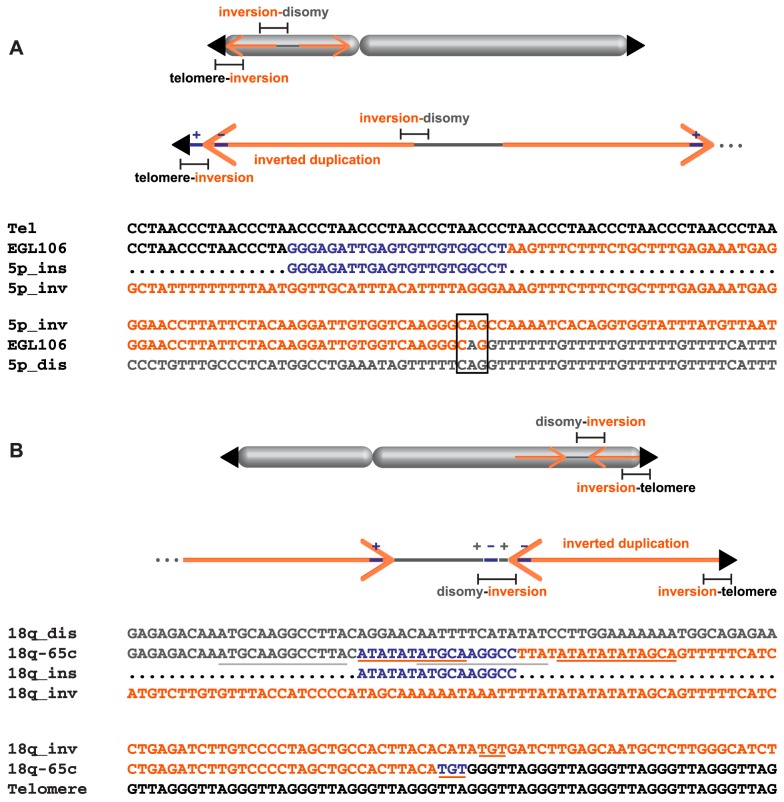
Complex junctions from EGL106 and 18q-65c. Insertion orientation (+/−) is indicated relative to the reference genome. (A) Alignment of telomere (black), inverted duplication (orange), inserted sequence (blue), and junction sequence (EGL106) from the telomere-inversion junction is shown above. The inverted duplication, disomic sequence (grey), and inversion-disomy junction sequence (EGL106) alignment is shown below. Microhomology at the junction is boxed. (B) Above, disomic, inserted, and inverted duplication sequences are aligned to the disomy-inversion junction sequence (18q-65c). Below, inverted duplication and telomere sequences are aligned to the inversion-telomere junction sequence (18q-65c). Inserted sequences and their neighboring homologous sequences are underlined.

Although most of the breakpoint junctions we sequenced were simple, transitioning from disomy to inversion, inversion to telomere, or inversion to translocation, five rearrangements had additional sequence inserted and/or inverted at the breakpoint junctions. EGL106 and 18q-6c had insertions at the inversion-telomere junctions of chromosomes 5p and 18q, respectively. Analysis of EGL106's inversion-telomere junction revealed a 22-bp insertion between the telomere and the inverted duplication. This sequence is identical to part of the inverted duplication, ∼600 bp from the end of the duplicated sequence (chr5:27,159,451–27,159,471). Interestingly, the inserted sequence is in the opposite orientation to that seen in the inverted duplication (Figure 6A). 18q-6c's inversion-telomere junction also has a small insertion that could be the product of replication slippage. Six basepairs of local junction sequence (TTTTTG) is inserted in the same orientation as the end of the inverted duplication (Figure S1).

SGTel015's disomy-inversion junction has a 4-bp insertion derived from the disomic side of the breakpoint. “CAAA” was inserted in the direct orientation between the inverted duplication of 5p and the disomic segment (Figure S1). 18q-65c's disomy-inversion junction also contains a short, 16-bp insertion: the first 11 bp are identical to sequence only a few bp away at the start of the inverted duplication, and the last 10 bp of the insertion are identical to nearby disomic sequence (Figure 6B). At the center of the 16-bp insertion, there are five bp (ATGCA) shared between both sides of the junction. Both halves of the insertion are in the same orientation relative to the disomic and inverted duplication segments. Insertions of local DNA sequence at breakpoints could occur via template slippage events [32], [33].

SGTel022's disomy-inversion junction contains a 70-bp insertion that lacks homology to nearby sequence on chromosome 2q (Figure S1). We aligned this sequence to the reference human genome using BLAT [34] and found all 70 bp to be mitochondrial in origin. The top alignment is homologous to positions 6513–6582 of the human mitochondrial genome, with all 70 bp aligning with 100% identity. The second-best alignment is homologous to a nuclear sequence of mitochondrial origin (numt) located on chromosome 1p that shares 97.2% sequence identity across 69 bp of the insertion sequence. Greater sequence homology to the mitochondrial genome than to existing numts is consistent with a new mitochondrial insertion that occurred at the time of inverted duplication formation [35]. A similar mitochondrial insertion has been described at the breakpoint of a balanced translocation between chromosomes 9 and 11 [36]. Like most mitochondrial insertions in primate genomes [37], the 70-bp insertion in SGTel022's junction lacks microhomology to the insertion site.

In addition to these five complex junctions, three out of 34 sequenced breakpoint junctions contain 1–3 bp of inserted sequence (Figure S1). Given the short insertion size, we cannot infer the origin of the inserted material. Most insertions are derived from the rearranged chromosome, usually within 1 kb of the breakpoint junction.

## Discussion

**Figure 7:**
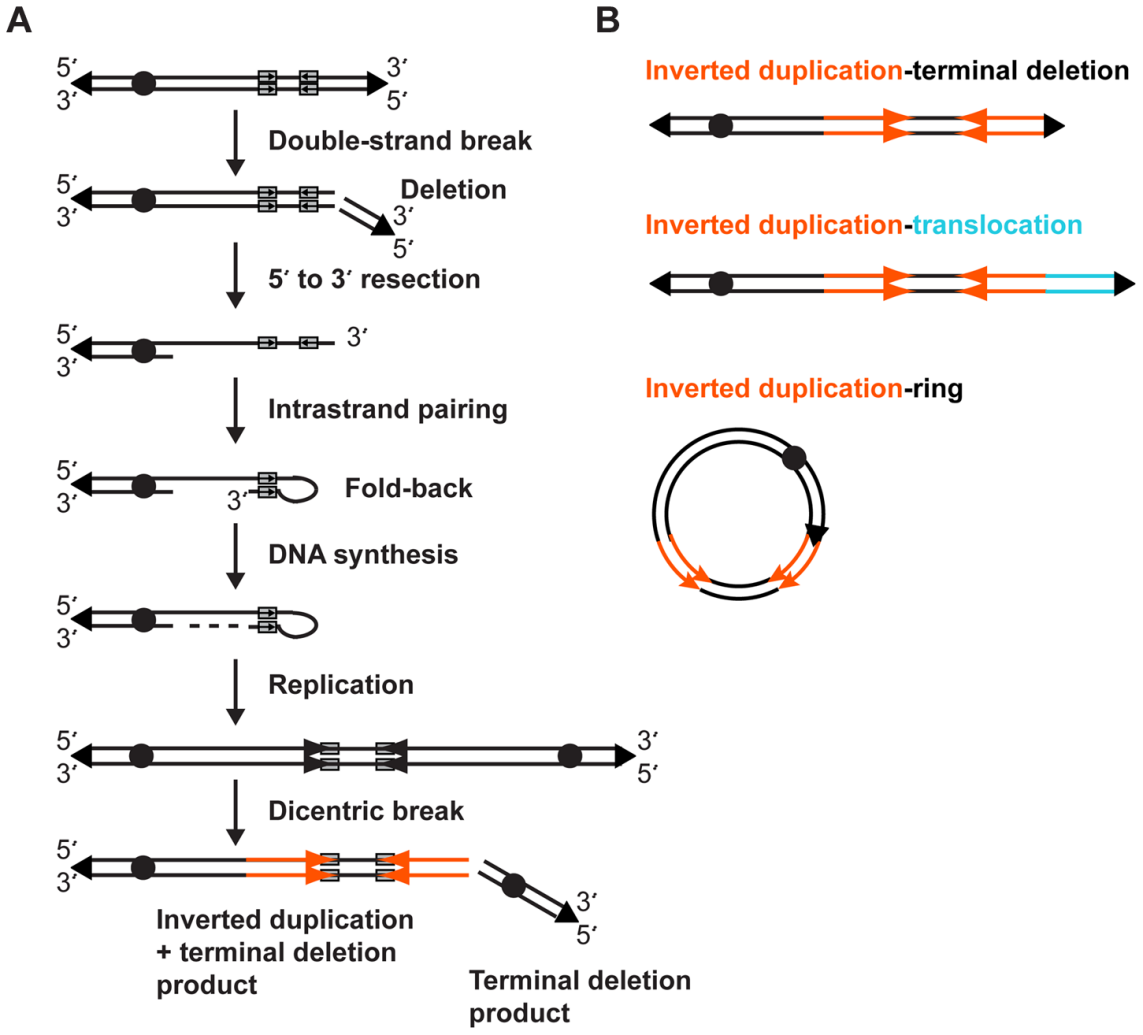
Fold-back model of inverted duplication formation. (A) 5′ and 3′ strands of the chromosome with telomeres (triangles) and centromere (circle) are shown. Short inverted sequences (grey rectangles with arrows) lie adjacent to the terminal deletion breakpoint. The inverted duplication mechanism occurs as described in the Discussion. The resulting inverted duplication is indicated by orange arrows. (B) After a breakage-fusion-bridge cycle, the inverted duplication chromosome may be repaired as a terminal deletion, translocation, or ring chromosome.

Sequence analyses of 34 breakpoint junctions in this study support a fold-back model of inverted duplication formation (Figure 7). We propose that an initial DSB generates a terminal deletion, then 5′-3′ resection of the free chromosome end creates a 3′ overhang that can intrastrand pair with itself, most often at a site of inverted sequence homology. DNA synthesis fills in the resected gap, creating a monocentric fold-back chromosome. Slippage during synthesis would produce templated insertions, derived from regions near the breakpoint [33]. Insertions could also arise via nonhomologous end-joining (NHEJ) or alternative NHEJ (alt-NHEJ) processes [38], especially for non-local insertions like the mitochondrial sequence in SGTel022. Insertions of 1–372 bp have been described in other inverted duplication breakpoints [9].

After DNA replication, the dicentric chromosome has a short disomic spacer in between the inverted sides of the chromosome, corresponding to the fold-back loop region. Such a dicentric chromosome is unstable during cell division, and after the BFB cycle(s), a second DSB between the two centromeres gives rise to two monocentric chromosomes: one with a terminal deletion and one with an inverted duplication plus a terminal deletion. The simple terminal deletion could acquire a new telomere or translocate with another free end; in either case there is no sign of the inverted duplication process in this chromosomal product. Terminal deletions are a relatively common type of CNV [29], and many could be formed through such a dicentric intermediate.

After dicentric breakage, there are at least three possible outcomes for the inverted duplication product (Figure 7B). Addition of a new telomere would produce a simple inverted duplication adjacent to a terminal deletion. End-joining between the free end of the inverted duplication and another chromosome would give rise to an inverted duplication translocation chromosome. Finally, fusion of the inverted duplication end and the other arm of the chromosome would produce a ring chromosome that harbors an inverted duplication. Though we did not analyze this type of chromosome rearrangement in this study, inverted duplication ring chromosomes consistent with this model have been reported [39], [40], [41]. Genotype analysis of inverted duplication ring chromosomes demonstrates the rings are derived from a single chromatid end, as predicted by our model, and not via a mechanism that requires recombination between homologous chromosomes [40]. All of these outcomes occur after a dicentric chromosome intermediate, so they may be subject to additional BFB cycles, resulting in additional copy number changes.

Telomere addition may occur through end-joining or through *de novo* synthesis of telomere repeats at the site of the DSB. Other terminal deletion telomere junctions include microhomology in some cases, and insertions in others [4], [29], [42], [43]. Similarly, three out of ten inversion-telomere junctions in our study had inserted sequences, and five junctions had 1–4 bp of microhomology with the (TTAGGG)n repeat (Figure S1).

The length of the fold-back loop will depend on the amount of DNA resection and the distance to the inverted sequence. In mammalian systems of induced DSBs, DNA resection is up to 1.3 kb; however, only rearrangements that preserve selectable markers are recovered, so those with greater resection lengths would be missed [33], [44]. Most of the disomic spacers described here are a few kb in size, within the range of DNA resection in other studies (Table 1 and Figure 5A). The amount of inverted homology required for intrastrand pairing at the fold-back loop is unknown. In our inverted duplications, we find 13 disomy-inversion junctions with 2–8 bp of microhomology, and three cases of ∼300 bp of sequence homology. Experimental inverted duplication systems have found similar lengths of inverted homology at breakpoint junctions. Tanaka et al. used 229-bp inverted repeats to stimulate inverted duplication formation in Chinese hamster ovary cells. Sequencing revealed “several nucleotides” of inverted microhomology at the breakpoint junctions [9]. In a yeast model of inverted duplication formation, as little as 4–6 bp of inverted homology was sufficient for fold-back [18]. Although short microhomologies are not sufficient to induce DSBs, they are likely to be important for intrastrand fold-back after DSBs.

We propose that the first step of duplication formation is a DSB that generates the terminal deletion. This exposes a free chromosome end that can intrastrand pair with itself to produce the characteristic inverted duplication and disomic spacer structure we observe in all junctions. Recently, Mizuno and colleagues described a HR-dependent mechanism of inverted duplication in fission yeast that does not require an initial DSB [45]. In this process, replication forks stalled at a replication-terminator sequence invade a nearby DNA strand at a site of inverted homology via NAHR. Resolution of the Holliday junction can produce dicentric chromosomes with inverted duplications and terminal deletions. Thus, it is possible that some human inverted duplications are initiated by replication fork stalling, rather than by a DSB. Fork stalling and template switching (FoSTeS) has been implicated in other complex breakpoints in the human genome that involve insertions and inversions [30], [46], [47], [48], [49], [50], [51], [52], and this process could explain complex junctions like those in 18q-65c and EGL106. However, in the fission yeast system, 150–1,200 bp of inverted homology was required for strand invasion [45]. The 2–8 bp of inverted microhomology we find at most inverted duplication junctions is not sufficient for NAHR, but it is possible that the ∼300 bp of homology between inverted *Alu*s or LINEs could be involved in HR-dependent strand invasion, similar to results from Mizuno et al.

Some have proposed a “U-type” exchange mechanism for human inverted duplication formation [6], [7]. In this model, pre-meiotic DSBs on sister chromatids of the same chromosome fuse to form a symmetric U-type structure. This dicentric chromosome is susceptible to breakage-fusion-bridge cycles, generating an inverted duplication chromosome and a terminal deletion chromosome. A key feature of this model is the absence of a disomic spacer between the inverted regions at the site of sister chromatid fusion. Lower-resolution studies will miss short disomic spacers, leading to the conclusion that U-type exchange is a common mechanism of inverted duplication formation [6], [7]. It is worth noting that we sequenced the disomy-inversion junctions of three subjects who were also included in the lower-resolution Rowe et al. (2009) study. EGL014 (Rowe 0152), EGL395 (Rowe 2998), and M397 (Rowe 9218) junctions have 3,428-bp, 2,914-bp, and 3,450-bp disomic spacers, respectively, which were not detected by the previous study [6]. This is not surprising since these samples were originally analyzed using arrays with probes spaced one every ∼75 kb [6], [53]. These discrepancies highlight the importance of sequencing breakpoint junctions when investigating chromosome rearrangement mechanisms.

Though we applied multiple experimental strategies to capture breakpoint junctions, some of the most complex junctions may have escaped detection due to large insertions or inversions that are difficult to infer from structural variation data. This is a common problem with CNV breakpoint studies, especially for those that include chromosome duplications [30], [54], [55], [56]. It is possible that segmental duplications at breakpoint junctions could have complicated junction sequencing; however, only one inverted duplication, SG_Tel_018, had a breakpoint near a segmental duplication. This segmental duplication is unlikely to be involved in SG_Tel_018's inverted duplication of chromosome 4q since the homology is shared between chromosomes 4 and 9, rather than the two regions of chromosome 4 involved in the rearrangement.

We were able to sequence half (34/68) of the attempted breakpoint junctions in our cohort (Table S1). This success was largely due to the integration of copy number data (high-resolution array CGH), DNA sequence analysis (PCR, SureSelect, NGS), and chromosomal localization of deletions and duplications (chromosome banding, FISH). Studies that rely on just one of these approaches will likely misinterpret chromosome rearrangements and confirm fewer breakpoint junctions. For example, M396's chromosome rearrangement was originally identified as an unbalanced translocation between chromosomes 10 and 18 by low-resolution array CGH and FISH, but high-resolution array CGH and sequencing of the inversion-translocation breakpoint revealed a 10-kb inverted duplication of chromosome 18 adjacent to the translocated segment from chromosome 10, consistent with an inverted duplication translocation chromosome. It is also important to point out that junction sequencing is dependent on the amount of DNA available for multiple sequencing strategies; for 22 inverted duplications, we exhausted the DNA sample (Table S1).

Microsatellite analysis of nine inverted duplications determined that the duplicated segment is always derived from the same chromosome as the original locus, not from the homologous chromosome. This indicates that the duplication arose through an intrachromosomal event, and points to intrastrand pairing within a sister chromatid. Other studies have also reported intrachromosomal inverted duplications [8], [40]. Copy number analyses of human blastomeres have revealed terminal deletions and duplications adjacent to terminal deletions involving the same chromosome end in different cells from the same embryo, consistent with the expected chromosomal products of our model [27], [28]. Furthermore, rare mosaic inverted duplication chromosomes have been described in lymphocytes and amniotic fluid [25], [26]. These data support a mitotic origin for nonrecurrent inverted duplications adjacent to terminal deletions. This is similar to the case for nonrecurrent CNVs that may be induced in mitosis by experimental conditions of replication stress [30], [57]. On the other hand, recurrent inverted duplications mediated by NAHR, such as the inv dup(8), likely originate during meiosis when homologous recombination occurs [23], [24]. Analysis of other recurrent chromosome rearrangements has shown that NAHR-mediated events are meiotic in origin [58].

All nine of the inverted duplications we analyzed for parent of origin occurred on a paternal allele. This is likely due in part to the paternal bias in rearrangements of chromosome 18. Heard et al. (2009) reported that 95/109 (87%) of *de novo* 18q deletions, duplications, and translocations are paternally derived [59]. Six of our inverted duplications arose on chromosome 18q and were part of the Heard study, two inverted duplications arose on chromosome 2q, and one occurred on chromosome 5p. Other studies have described maternal and paternal origins of inverted duplications [4], [8], [60], [61], [62], [63], [64], [65]. These data argue against a parent-of-origin bias for inverted duplications overall.

Inverted duplications almost always occur *de novo*. In our cohort, 25/26 inverted duplications were not present in parents in either a balanced or unbalanced state. Other studies of inverted duplications find similar inheritance patterns [6], [7]. Furthermore, analysis of human blastomeres detects inverted duplications with terminal deletions as new events in the developing embryo [27], [28]. Together, these data suggest that the inverted duplication and terminal deletion occur in a single step, rather than as a progression from a balanced rearrangement in an unaffected parent to unbalanced inheritance in an affected child. This is an important finding when considering recurrence risk for inverted duplication formation in genetic counseling.

Our large-scale breakpoint analysis has determined the genomic structure and CNV formation mechanism for human inverted duplications. Disomic spacers between inverted regions point to a fold-back step, and short inverted sequences at breakpoint boundaries are consistent with fold-back looping that occurs after the DSB and DNA resection steps of the chromosome rearrangement. Complex breakpoints may arise via template insertions during DNA synthesis or via alt-NHEJ. These data support a fold-back mechanism for nonrecurrent inverted duplications.

## Materials and Methods

### Ethics statement

We received peripheral blood and/or DNA samples from subjects with pathogenic CNVs and their parents. Samples were ascertained from the Emory Genetics Laboratory (EGL), Signature Genomic Laboratories (SG), the Chromosome 18 Clinical Research Center (18q-), and the Martin laboratory (M). See Table S1 for details. This study was approved by the Emory University Institutional Review Board.

### High-resolution array CGH

Chromosome rearrangements were originally analyzed in clinical cytogenetics laboratories with different array CGH platforms, subtelomeric FISH assays, and/or G-banded chromosome analysis. Array CGH results were confirmed by chromosome analysis or FISH in diagnostic laboratories using standard methodologies. We confirmed all chromosome rearrangements via custom high-resolution array CGH.

We designed custom 60k CGH arrays with oligonucleotide probes targeted to previously identified breakpoints with a mean probe spacing of one probe per 200 bp. Oligonucleotide arrays were designed with Agilent's eArray program (https://earray.chem.agilent.com/earray/). Custom array designs (AMADID numbers) are listed in Table S1. DNA extraction from peripheral blood and cell lines, microarray hybridization, array scanning, and breakpoint analysis were performed as described previously [29]. Array CGH data have been submitted to the NCBI Gene Expression Omnibus (GEO) database under accession number GSE45395 (http://www.ncbi.nlm.nih.gov/geo/).

### SureSelect and NGS

We designed SureSelect libraries to target the 40 kb flanking CNV breakpoints mapped by high-resolution array CGH. SureSelect target enrichment baits were designed using the “Bait Tiling” option in eArray. 120-bp baits were tiled with 3x coverage, 20-bp allowable overlap, and a centered design strategy. Electronic Library ID (ELID) #0349851 targeted breakpoint regions from 18q-186c and M397; ELID #0368031 targeted breakpoint regions from 18q-26c, 18q-119c, 18q-62c, M396, EGL044, EGL398, EGL399, and EGL074 (Table S1).

SureSelect capture and Illumina HiSeq sequencing were performed at Hudson Alpha Genomic Services Lab (http://www.hudsonalpha.org/gsl/). After NGS, we aligned 100-bp paired-end reads from fastq files to the GRC37/hg19 reference genome using Burrows-Wheeler Alignment (BWA) tool 0.5.9 [66] and identified misaligned pairs using the SAMTools 0.1.18 filter function [67]. Paired-end reads that aligned to the reference genome too far apart, too close together, in the wrong orientation/genome order, or to different chromosomes were clustered to predict structural variation, as described [68]. We identified split reads using CIGAR scores of the aligned reads and inspected junctions manually using Integrative Genomics Viewer (IGV) [69]. Using this approach, we successfully captured M397's inversion-translocation breakpoint, EGL074's disomy-inversion junction, and EGL044's disomy-inversion and inversion-telomere junctions. These junctions were confirmed by PCR and Sanger sequencing. Sequence data from SureSelect experiments have been deposited at the Sequence Read Archive (https://submit.ncbi.nlm.nih.gov/) under accession number SRP032751.

### Breakpoint amplification and sequencing

We performed long-range PCR to amplify breakpoint junctions inferred from high-resolution array CGH following conditions described previously [29]. PCR primers are listed in Table S3. We optimized reactions by adjusting the MgCl concentration (1 mM–3 mM) or by adding Betaine (0.7–2.0 M), DMSO (1–10%), and/or Tween 20 (0.5–2%). We designed PCR primers to cross the two sides of the inverted duplication junction (including the disomic spacer), the disomy-inversion junction, the inversion-telomere junction, and the inversion-translocation junction, as appropriate. For inversion-telomere junctions, we designed a primer complementary to the inverted duplication side of the junction and paired this primer with one of two telomere primers, 5′-CCCTAACCCTAACCCTAACCCTAACCCTAA-3′ or 5′-TATGGATCCCTAACCCTGACCCTAACCC-3′ [42].

The disomy-inversion junction from 18q-26C was amplified via inverse PCR. A BsrDI restriction site is located ∼2 kb proximal to the distal end of the duplication, but is absent from the predicted spacer region. Genomic DNA (5 µg) from 18q-26C and a normal control was digested following the manufacturer's protocol (NEB #R0574S; 1 h at 65°C, 20 min at 80°C, and store at 4°C). Digested DNA was purified with a QIAquick Purification Kit (#28106) following the manufacturer's protocol. Blunt ends were created using T4 DNA Polymerase (NEB #M0203S) in 1X NEBuffer 2, supplemented with 100 µg/ml BSA and 100 µM dNTPs in a 50-µl reaction incubated 15 min at 12°C. The reaction was stopped with 1 µl of 0.5 M EDTA heated to 75°C for 20 min. Blunt-end fragments (≤50 ng DNA per 20 µl ligation reaction) were circularized and ligated with T4 DNA Ligase (Quick Ligation Kit, NEB #M2200L) for 5 min at room temperature. We performed PCR on circularized template DNA using outward-facing primers and standard PCR conditions.

PCR-amplified junctions were Sanger sequenced (Beckman Coulter Genomics, Danvers, MA). We aligned DNA sequences to the human genome reference assembly (GRC37/hg19) using the BLAT tool [34] on the UCSC Genome Browser (http://genome.ucsc.edu/). Disomy-inversion junctions from 18q-233c, SGTel014, and EGL104 aligned to interspersed repeats (Figure S2). Other junction sequences are described in Figure S1. Breakpoint junction sequences have been submitted to GenBank under project number 1611902. Accession numbers are listed in Table S1.

### Microhomology simulation

To estimate the amount of inverted microhomology expected by chance at disomy-inversion breakpoints, we simulated 1,000 spacers in the human genome. We used the random number function and a custom Perl script to generate sequence coordinates for sequences less than 70,466 bp long (maximum sequenced spacer length) and within 5.5 Mb from the chromosome end (median terminal deletion size) from random chromosomes. Disomic spacers in the simulated dataset are between 811 bp and 70.5 kb long (mean = 36.1 kb). We downloaded each disomic spacer sequence from the Ensembl database and counted the bp of microhomology between the 3′ end of the spacer and the reverse complement of the 5′ end, allowing for zero mismatches using Perl regular expressions. The frequency of 0–8 bp of simulated inverted microhomology compared to observed microhomology is shown in Figure 5B.

To compute an empirical p-value based on these simulations, we first noted that 134 of 1,000 simulations had microhomology of ≥2 bp (the minimum-sized microhomology in the 13 disomy-inversion junctions). We then used simple combinatorics to count 1) the number of different 13-junction groups that could be formed from 134 simulated junctions, and 2) the number of different 13-junction groups possible from 1,000 simulated junctions. We computed our empirical p-value as the ratio of these values: 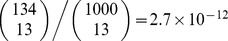; this value is a simulation-based estimate of the proportion of 13-junction groups that would have ≥2 bp of microhomology for all 13 junctions by chance alone.

### Microsatellite analysis

Microsatellite markers within the deleted and duplicated regions were selected from the UniSTS database (http://www.ncbi.nlm.nih.gov/unists; Table S2). We used the Type-it Microsatellite PCR Kit (Qiagen, Valencia, CA) and primers labeled with 6-carboxyfluorescine (6-FAM) or hexachloro-fluorescein (HEX) (Integrated DNA Technologies, Coralville, Iowa). Amplification was performed in 25-µl volumes with 50 ng of DNA template and 0.2 µM of each primer in a multiplexed reaction. The PCR cycles were 95°C for 5 min, then 26 cycles at 95°C for 30 s, 58°C for 90 s, 72°C for 30 s, with a final extension of 60°C for 60 min. We ran amplicons on a 16-capillary Applied Biosystems 3130XL Genetic Analyzer with a GeneScan 500 size standard. GeneMarker Software v1.95 (Soft Genetics, LLC, State College, PA) was used to size the alleles to the nearest bp and determine peak heights.

## Supporting Information

Figure S1 Alignment of breakpoint regions to the reference genome. Microhomology is highlighted in yellow. Insertions are shown in blue.
(DOCX)

Figure S2 Disomy-inversion junction sequences from 18q-233, SGTel014, and EGL104 span repeats. The junction sequence from 18q-233c aligns to a L1PA2 repeat on the disomy side (chr18:68,483,852–68,484,316) and a L1Hs repeat on the inverted duplication side (chr18:68,413,423–68,414,115). The SGTel014 junction aligns to an *Alu*Sx1 on the disomy side (chr2:239,914,588–239,914,988) and an *Alu*Sq2 on the inverted duplication side (chr2:239,905,672–239,906,058). The EGL104 junction aligns to an *Alu*Y on the disomy side (chr9:10,503,833–10,504,408) and an *Alu*Y on the inverted duplication side (chr9:10,518,536–10,519,097). Genomic coordinates are based on the GRC37/hg19 build of the human genome assembly.
(DOCX)

Table S1 Breakpoint junctions as determined by array CGH and sequencing. Genomic coordinates (GRC37/hg19) are shown for breakpoints mapped by low-resolution array (green), high-resolution array (red), or sequenced junction (black). The number of disomy-inversion, inversion-telomere, and inversion-translocation junctions sequenced per rearrangment are shown. Spacer sizes are listed for sequenced disomy-inversion junctions. Spacers without a sequenced Dis-inv junction were measured from most distal duplicated probe to the most proximal deleted probe on the array. Please note, there is a 245-kb gap in probe coverage for the region corresponding to SG_Tel_025's spacer region. The spacer calculation of 286 kb is likely an overestimate.
(XLSX)

Table S2 Microsatellite analysis of deleted and duplicated alleles. Alleles with peak heights representing the duplication are indicated by an asterisk (*). The inheritance of microsatellites revealed paternal duplications (Pat dup), paternal deletions (Pat del), paternal translocations (Pat trans), or uninformative markers (U). Double bars separate duplication and deletion regions. In three families, one parent was not available for testing (-). In these cases, we infer the origin of the duplication based on the alleles present/absent in the parent who was tested. For 18q-199c and EGL106, only maternal samples were tested. The duplicated markers are derived from the missing (paternal) alleles, and the maternal allele is retained in the deleted region. Since we did not test fathers, it is possible albeit unlikely that mothers and fathers have the same genotype in the deleted region, making deletion markers uninformative. Retention of the maternal allele in the deletion region is consistent with a paternal deletion. We have indicated these caveats as (Pat del?).
(DOCX)

Table S3 PCR primers used to amplify 34 breakpoint junctions. The corresponding subject, chromosome end, and GenBank accession number are listed for each primer pair.
(XLSX)
