# Supplementary material for: Large Inverted Duplications in the Human Genome Form via a Fold-Back Mechanism
Source: PLoS Genet. 2014 Jan 30;10(1):e1004139. doi: 10.1371/journal.pgen.1004139 (PMC3907307; doi:10.1371/journal.pgen.1004139)
Supplement: Figure S1 — Alignment of breakpoint regions to the reference genome. Microhomology is highlighted in yellow. Insertions are shown in blue. (DOCX) [file pgen.1004139.s001.docx]

**Disomy-inversion junctions**

2q_disomy

CCAGCCTGGgcgacagagcgagactccgcctcaaaaaaaaaaagaaaagaaatatggtgcttggttcttaattttgcagcattcaggac

SGTel022

CCAGCCTGGTGCTGGCATCACTATACTACTAACAGACCGCAACCTCAACACCACCTTCTTCGACCCCGCCGGAGGAGGAGACATGATGA

Mitochondrial insertion

.........TGCTGGCATCACTATACTACTAACAGACCGCAACCTCAACACCACCTTCTTCGACCCCGCCGGAGGAGGA..........

2q_inv

cacacgatggaatagtactcagccttcttaaaggctgcatattctaatattcagggacagaaattctaatatctgctacGACATGATGA

2q_dis CTAATTTTATGTCCTTATACTTATTTTCTCTGAAACCTAATGCTAGTTTATATCACCTTGC

EGL044 CTAATTTTATGTCCTTATACTTATTTTCTCTTTGAAAACGCTAATAACATACAGAGCCCTG

2q_inv AGGAACATTGATAAATACAATGACTCAGTCTTTGAAAACGCTAATAACATACAGAGCCCTG

2q_dis AGAGGAGCCTTGTTTCCTATTAAACTGGGATCTCTGGCAACCGAGAGGTGCCTGGGGGTGC

EGL395 AGAGGAGCCTTGTTTCCTATTAAACTGGGAAACCTGAGCCCCATCCATTCCTCTTAAACCC

2q_inv TCTGCAGCGCTGTCTTCCCTGAGACTTTGCTACCTGAGCCCCATCCATTCCTCTTAAACCC

4p_inv TGAGCCACCGCGCCCAGCCAGTTCTTGGACCTTTCTAAAAATGAGTCTTATTCTGCTTCCC

EGL014 TGAGCCACCGCGCCCAGCCAGTTCTTGGACCTGTCTTTCTGATGTCTCTTTCCCAAGCCCC

4p_dis GATGCAGGCGTGCAGGCACTGCACTGTCTTCTGTCTTTCTGATGTCTCTTTCCCAAGCCCC

4p_inv AAGCTCACATACTAAATGTGTAACCTTGGACAAGTCACTTAGCTTTTCCCAAACTGGTTAA

SGTel013 AAGCTCACATACTAAATGTGTAACCTTGGACACCTGCAGTCCGTAGGCCAAGTCTAGCCTG

4p_dis GAGTAAAAGGTGAGGAAGCAGAGGTCACGTCACCTGCAGTCCGTAGGCCAAGTCTAGCCTG

5p_inv TATCTGTTGTTTGTTTTTGCTTTTTGCTGTTCTAATACTTTCTCCTGAACTTAAACTGGC

SGTel015 TATCTGTTGTTTGTTTTTGCTTTTTGCAAAACAAAGTTCTTAGGAAATCTGTAAGTTTAG

5p_ins ..........................CAAA..............................

5p_dis ATGTTCAGTAACCAATCTACAGGGCCTGCCACAAAGTTCTTAGGAAATCTGTAAGTTTAG

5p_inv GGAACCTTATTCTACAAGGATTGTGGTCAAGGGCAGCCAAAATCACAGGTGGTATTTATGTTAATC

EGL106 GGAACCTTATTCTACAAGGATTGTGGTCAAGGGCAGGTTTTTTGTTTTTGTTTTTGTTTTCATTTG

5p_dis CCCTGTTTGCCCTCATGGCCTGAAATAGTTTTTCAGGTTTTTTGTTTTTGTTTTTGTTTTCATTTG

9p_inv CACAGTTTTTGGCTGTCCTGGGAACAACAATCTAGTGCAACTCCAGCAATGTGAGTTATAG

EGL074 CACAGTTTTTGGCTGTCCTGGGAACAACAATACTTTGCCATAGAATTCACATCTTTGTTTT

9p_dis TGTAGTATGCAAAGGTCATTTGTAAACTCATACTTTGCCATAGAATTCACATCTTTGTTTT

9p_inv ATATACCGTCTATGCCCTTAACTACCCCTGCCACTGGATTTTTTTAAGTGGAGCAAATTA

M397 ATATACCGTCTATGCCCTTAACTACCCCTGATCAATGATTTGGAGCACCTTTTCATATGT

9p_dis ATAATAGTTTTGATTTGCATTTCTCTGATGATCAATGATTTGGAGCACCTTTTCATATGT

13q_dis GGCTCTTGGGAATTTCGTTTTGCTTTTACGGGAATGTGGCATGGTCAGCGGTGGCCATCAG

SGTel019 GGCTCTTGGGAATTTCGTTTTGCTTTTACGACTGTCCATCAGACAGGACAGTCGTGCCTTT

13q_inv CAAAATGCCCATTCCCAAAGGCTTCTGCATCCTGTCCATCAGACAGGACAGTCGTGCCTTT

18q_dis GTATCGCCAGGTGTCCTGTTTCTGCGTTGTTCCCATGTTTTACTTCCTCACTGTTTTGCCT

18q-207c GTATCGCCAGGTGTCCTGTTTCTGCGTTGTTATCTCATTCCATCCTTCCTGTAAACCTATA

18q_inv GAAGAACTATTATAAATGCTTTCCATGGATTATCTCATTCCATCCTTCCTGTAAACCTATA

18q_dis CTGATGTTTGTGCAGAATGACTACTTTATACCGTACAGAAGGAGCACACTCTCTTCTTGTG

EGL099 CTGATGTTTGTGCAGAATGACTACTTTATACCGGCTACCCTCACCCTGCTCCCAGTCCAAT

18q_inv TCTGGCTAAATTCTAGTGCCAGGTGGCTCTACGGCTACCCTCACCCTGCTCCCAGTCCAAT

18q_dis GATGGTTTCCTTTGGGTTTTTTACCC_CTGTTCAATGTACCAATTTATAATCCTAT

18q-26c GATGGTTTCCTTTGGGTTTTTTACCCACTGTCACTTGTGGAAACGCAACTACACAG

18q_inv GTTGACCCTGTATATTCAACAATTACACTGTCACTTGTGGAAACGCAACTACACAG

18q_dis TATTGCCTTTTATCTGAAAAAAGATTAAATTATATTAGATTCATCTATGGCAATTTTCTTCC

18q-6c TATTGCCTTTTATCTGAAAAAAGATTAAATTCCATTCCTGGTTGTGTATCCAAAAGAATTGA

18q_inv AGAAACAGAGTTATCATATGATTCAGCAATTCCATTCCTGGTTGTGTATCCAAAAGAATTGA

18q_dis TCGGAATCGGGATGCTGATGGCTCCAGGTCTCTGGCTCTAGACAGTCTACTCATCCCCGT

18q-223c TCGGAATCGGGATGCTGATGGCTCCAGGTCTTATTTCATTTTGTTTGTTGGCGACATTTA

18q_inv TTGCCTTGGCCAAAAAATACATCCCAGGTCTTATTTCATTTTGTTTGTTGGCGACATTTA

18q_dis GAGAGACAA**ATGCAAGGCC**TTACAGGAACAATTTTCATATATCCTTGGAAAAAAATGGCAGAGAA

18q-65c GAGAGACAA**ATGCAAGGCC**TTACATATATATGCAAGGCCTTAT**ATATATATAGCA**GTTTTTCATC

18q_ins .......................**ATATATATGCAAGGCC**..........................

18q_inv ATGTCTTGTGTTTACCATCCCCATAGCAAAAAATAAATTTTAT**ATATATATAGCA**GTTTTTCATC

18q_dis AAAAATAGTTTGACCTGTTTAGATTCAGGGGTATGTGTGCAGGTTTGTTACATTGTGTGATG

18q-139c AAAAATAGTTTGACCTGTTTAGATTCAGGGGTTTTATAAATTCCTCAATGTTAAGAGATTTG

18q_inv GAGTGCTTTAGGAATTAACTTTTACTTGGGGTTTTATAAATTCCTCAATGTTAAGAGATTTG

18q_dis AGCAAGCAAGACTAAAATTTTAACATATACAGCAATAACACTGACATCTGTCTGTCTTAGC

18q-107c AGCAAGCAAGACTAAAATTTTAACATATACAGATTATATTTAGAGTTAGGGAAAATAATTC

18q_inv GAATAGCAAATTGAAATTTTCATTATGCTGAGATTATATTTAGAGTTAGGGAAAATAATTC

**Inversion-telomere junctions**

2q_inv TCTTATTTTTCCATTTCTGATTGATCTAGCACTATCATCCATGTTTATGCATATTATTTAAAAT

EGL044 TCTTATTTTTCCATTTCTGATTGATCTAGCAGGGTTAGGGTTAGGGTTAGGGTTAGGGTTAGGG

Tel AGGGTTAGGGTTAGGGTTAGGGTTAGGGTTAGGGTTAGGGTTAGGGTTAGGGTTAGGGTTAGGG

Tel CCTAACCCTAACCCTAACCCTAACCCTAACCCTAACCCTAACCCTAACCCTAACCCTAACCCTAACCCTAACCCTAA

EGL106 CCTAACCCTAACCCTAGGGAGATTGAGTGTTGTGGCCTAAGTTTCTTTCTGCTTTGAGAAATGAGAGAAAATAGTAC

5p_ins ................GGGAGATTGAGTGTTGTGGCCT.......................................

5p_inv GCTATTTTTTTTTAATGGTTGCATTTACATTTTAGGGAAAGTTTCTTTCTGCTTTGAGAAATGAGAGAAAATAGTAC

Tel CCCTAACCCTAACCCTAACCCTAACCCTAACCCTAACCCTAACCCTAACCCTAACCCTAACCCTAACCCTAACCCTA

EGL104 CCCTAACCCTAACCCTAACCCTAACCCTAAATAGCACTAAATGCCCACAAGAGAAAGCAGGAAAGATCTAATATTGA

9p_inv ACATTCAAAGCAGTGTGTAGAGGGAAATTTATAGCACTAAATGCCCACAAGAGAAAGCAGGAAAGATCTAATATTGA

18q_inv GGCAAATCCACAGAGACAGAAAGTAAATCAGTGGTTGCCAGGGCCTGGGAGGAAGGGGAATGAG

18q-207c GGCAAATCCACAGAGACAGAAAGTAAATCAGTGGTTAGGGTTAGGGTTAGGGTTAGGGTTAGGG

Tel AGGGTTAGGGTTAGGGTTAGGGTTAGGGTTAGGGTTAGGGTTAGGGTTAGGGTTAGGGTTAGGG

18q_inv TTACTATTCAACACAGCACTGGAAGTTTTAGCCACTGCAATCAACCAAGAAAATAAATGTAA

18q-26c TTACTATTCAACACAGCACTGGAAGTTTTAGGTTAGGGTTAGGGTTAGGGTTAGGGTTAGGG

Tel GGTTAGGGTTAGGGTTAGGGTTAGGGTTAGGGTTAGGGTTAGGGTTAGGGTTAGGGTTAGGG

18q_inv CTTCACAGAAATAGCCCTCCAGCATTTTTGTAGGTATGAAGATAATTTAATTCTCCATAGGG

18q-6c CTTCACAGAAATAGCCCTCCAGCATTTTTGTTTTTGTTAGGGTTAGGGTTAGGGTTAGGGTT

18q_ins ..............................TTTTTG..........................

Tel TTAGGGTTAGGGTTAGGGTTAGGGTTAGGGTTAGGGTTAGGGTTAGGGTTAGGGTTAGGGTT

18q_inv ATTGTATAAAAATTGCAATGTTTGGATTTCCAGGTAAAATCTTTTCACCGCAGCTCTTG

18q-34c ATTGTATAAAAATTGCAATGTTTGGATTTCCAGTAGGGTTAGGGTTAGGGTTAGGGTTA

Tel GGTTAGGGTTAGGGTTAGGGTTAGGGTTAGGGTTAGGGTTAGGGTTAGGGTTAGGGTTA

18q_inv CTGAGATCTTGTCCCCTAGCTGCCACTTACACATATGTGATCTTGAGCAATGCTCTTGGGCATCTGTTTGTTCATC

18q-65c CTGAGATCTTGTCCCCTAGCTGCCACTTACATGTGGGTTAGGGTTAGGGTTAGGGTTAGGGTTAGGGTTAGGGTTA

Tel GTTAGGGTTAGGGTTAGGGTTAGGGTTAGGGTTAGGGTTAGGGTTAGGGTTAGGGTTAGGGTTAGGGTTAGGGTTA

18q_inv GCCTGTAATCCCAACACTTTGGGAGGCTGAGATGAGCCGATCACCTGAGGTCAGGAGTTCAA

18q-223c GCCTGTAATCCCAACACTTTGGGAGGCTGAGGTTAGGGTTAGGGTTAGGGTTAGGGTTAGGG

Tel GGTTAGGGTTAGGGTTAGGGTTAGGGTTAGGGTTAGGGTTAGGGTTAGGGTTAGGGTTAGGG

18q_inv ATGTTATCACTCTCAGATATTGCCAAGTGTTTTCCAAAGTGGCTGCAATTATTCACCCTCCAT

SGTel009 ATGTTATCACTCTCAGATATTGCCAAGTGTTTAGGGTTAGGGTTAGGGTTAGGGTCAGGGTTA

Tel TTAGGGTTAGGGTTAGGGTTAGGGTTAGGGTTAGGGTTAGGGTTAGGGTTAGGGTTAGGGTTA

**Inversion-translocation junctions**

7q_inv TTATTTATTTATTTATTTAGACAGAGTCTCACTCTGTTGCCCAGGCTGGAGTGCAGTGGGGCATGATCTCGGCTC

EGL399 TTATTTATTTATTTATTTAGACAGAGTCACACTCTGTGTTCAATTATCTGGAAAATTGATCTGATCCCCGACATT

8p TAACCCCATTTAACTCTAACTCATCCGTCAGCTCTGAGTTCAATTATCTGGAAAATTGATCTGATCCCCGACATT

20p CCTGCATCTCAGGGATAAACCACACTTGATCACGGCATGTGATCTTTTCACTGTGTTGTTG

M397 CCTGCATCTCAGGGATAAACCACACTTGATAACTTTATTTTTTTCTTTTCTCCTGACACTT

9p_inv AACAAAAATATTTATCATTACCAAGATAATAACTTTATTTTTTTCTTTTCTCCTGACACTT

18q_inv ATGTGTGGCCATGTGCCGGGTGAGGCTGCCATTGGTGGTGAAGGAAGCATTGCACACAC

M396 ATGTGTGGCCATGTGCCGGGTGAGGCTGCCACGTAATGCGGTCGCTAAGAAGCCCGCAG

10q GCCGACCAGGCTTTTCTTGCCCGCTAGTCCACGTAATGCGGTCGCTAAGAAGCCCGCAG
