# Supplementary material for: Large Inverted Duplications in the Human Genome Form via a Fold-Back Mechanism
Source: PLoS Genet. 2014 Jan 30;10(1):e1004139. doi: 10.1371/journal.pgen.1004139 (PMC3907307; doi:10.1371/journal.pgen.1004139)
Supplement: Figure S2 — Disomy-inversion junction sequences from 18q-233, SGTel014, and EGL104 span repeats. The junction sequence from 18q-233c aligns to a L1PA2 repeat on the disomy side (chr18:68,483,852–68,484,316) and a L1Hs repeat on the inverted duplication side (chr18:68,413,423–68,414,115). The SGTel014 junction aligns to an AluSx1 on the disomy side (chr2:239,914,588–239,914,988) and an AluSq2 on the inverted duplication side (chr2:239,905,672–239,906,058). The EGL104 junction aligns to an AluY on the disomy side (chr9:10,503,833–10,504,408) and an AluY on the inverted duplication side (chr9:10,518,536–10,519,097). Genomic coordinates are based on the GRC37/hg19 build of the human genome assembly. (DOCX) [file pgen.1004139.s002.docx]

>18q-233_jxn

TGAAAAGATTAATAAATTTGATAAACCCCTTGCCAGGCTTCCAGGGGAGGGAAAAAACAAAAAGAAGACACAAGTTGAAAATACAAAAAAAAGAAGGAACGTAACTACTGATCCAATAGATGTTAAAAGAATGTTAATGGTTCATGTCCTTTGTAGGGACATGGATGAAATTGGAAACCATCATTCTCAGTAAACTATCGCAAGAATAAAAAACCGAACACCGCATATTCTCACTCATAGGTGGGAATTGAACAATGAGATCACATGGTCACAGGAAGGGGAATATCACACTCTGGGGACTGTGGTGGGGTGTGGGGAGGGGGGAGGGGTAGCATTGGGAGATATACCTAATGCTAGATGACGAGTTAGTGGGTGCAGCACACCAGCATGGCACATGTATACATATGTAACTAACCTGCGCAATGTGCACATGTACCCTAAAACTTAGAGTATAATAAAAAAAAATAATAATAATAATAGCACCCCATTTACCATGTAAGGCTCATCACAACATATTTCCCATCTGGTTTCTCTAACTCCAGTGCCCTATTTCTCCCCACAGAAGATAATGACCCATCTCTATTTCCATCACTCCTGCCAGCCTTGGCCTGCCAAGGAGACCCACCACTGAACTTCTTAGTGATGCTGGTGACCCTATTGCCACCAAACCATTAATGCCTTTTATAAATGCTGCATCTAGGCTCTCTTGGGGTTTTCTGGTCTTACCTGATACAGAAACTAGAAGCATGGTGAGTACAAGTACCCATGAGAGGAGATAGGGGCATCTCCTGAGATAGATGGCTGTTGCGTTGCAGGGGAGAGGTCTCTAGCATT

>SGTel014_jxn

CCTAACTCCTGTTGCACTTCAGGAAAGCACAGGTCCCCAGGCTCTGCCTGAAGGATTTTT

TTTCGTGGGAATTTACTCCTTAGATATACCCCACTATACAAAAACACCTGTATATAAAGT

TATCTAGTCGGGCACCGTGGCTTATGCCTGCAATCTCAGCACTTTGAGAGGCTGAGGCGG

GTGGATCACTTGAGGTCAGGAGTTCGAGACCAGCCTGGCCAACATGGTGAAACCCCATCT

CTACTAAAAAAATACAAAAATTAGCCAGGTGTAGTGGCAGGCACCTATAATCCCAGCTAC

TTGGGAGGCTGAGGCAGGGAGAATTGCTTGAACCCAGGAGGTAGAGGTTGCAGTGAGCAG

AGATCGCACTGCTGCACTCCAGCCTGGGTGACAGAGCAAAGGGTATAATAATCAAATCAG

GCCCTCGGGGGGCCAAGGCAGGAGAATCGCTTGAACCTGGGAGGCAGAGGTTGCAGTGAG

CCGAGATTGCGCCATTGCACTCCAGCCTGGGTGACAAGAGCGTGACTTCGTCTCAAAACA

AACAAACTAACCAAAAACAAAGCTCTGGCCTGCAACCTGCGGCAACCAGCCCCGGAAACC

CACCCCTTTCCTACAGTGACCAGCCCCGAGGCCAGCTTGCTGTCAAT

>EGL104_jxn

TCCTGCAGGTTTAACGAATTCGCCCTTCTCGTCTGACAATGAGGATATGCTGCTGGATTT

GTAGTATCAGAAAGCTGGGAGGCAAAAGGTAAGAGTTAGCTGAAAATAGGAGATAAGAGT

GCAGGTTTCTTCTGACCATTAAAAAAATAATCTACCCAGAAAAAGAAAATTAGCATGGCA

TCTTCTTGCCCTGTTCATTATCTTTTATGTATGAAGAGGATTTATTTTAATTATTTTGAA

ATGGCTGTTAAAACAATTTCAATTATTATTTAAAAAAAAAAAACAATGTAAGGCTGAGCG

CGGTGGCTCACGCCTGTAATCCCAGCACTTTGGGAGGCCAAGGCGGGCAGATCACGAGGT

CAGGAGATCAAGACCATCTTGGCTAACACGGTAAAACCCTGTCTGTACTAAAAATACAAA

AAATTAGCCGGGTGTGGTGGCGGGCGCCTGTAGTCCCAGCTACTCCGGAGACTGAAGCAG

GAGAATGGCGTGAACCCGGGAGGCAGAGCTTGCAGTGAGCCGAGATCGCGCCACTGCACT

CCAGCATGGGGCACAGAGCGAGACTCTGTCTCNNAAAAAAAAAAAAAAAAAAAAAAAAAA

AAAAGGAGGATGTACGGGGAAAGTGTCATTGAATGGCGATTCTTGAGTCTAAAGCCAAAG

GCGGTAACTATTGCTGTAACAGGTGTGATATATGCATCGAACTCTCTCCTATCTACATGT

ACACAAACAGTCTTAAAATGAACATCACTGCAAGCCAGTATTATGATTAATAGTTAATTT

GACTCTCTAGACTTTTTATTTAAATATCTAGATAAAATCCACCCACAGCTCCAAGTGACA

GAACACTTCGGGTTACTCTGCTTAGTAGGCTTCTGAAGGGCGAATCGCGGCCGCTAAATT
