# Supplementary material for: Large Inverted Duplications in the Human Genome Form via a Fold-Back Mechanism
Source: PLoS Genet. 2014 Jan 30;10(1):e1004139. doi: 10.1371/journal.pgen.1004139 (PMC3907307; doi:10.1371/journal.pgen.1004139)
Supplement: Table S2 — Microsatellite analysis of deleted and duplicated alleles. Alleles with peak heights representing the duplication are indicated by an asterisk (*). The inheritance of microsatellites revealed paternal duplications (Pat dup), paternal deletions (Pat del), paternal translocations (Pat trans), or uninformative markers (U). Double bars separate duplication and deletion regions. In three families, one parent was not available for testing (-). In these cases, we infer the origin of the duplication based on the alleles present/absent in the parent who was tested. For 18q-199c and EGL106, only maternal samples were tested. The duplicated markers are derived from the missing (paternal) alleles, and the maternal allele is retained in the deleted region. Since we did not test fathers, it is possible albeit unlikely that mothers and fathers have the same genotype in the deleted region, making deletion markers uninformative. Retention of the maternal allele in the deletion region is consistent with a paternal deletion. We have indicated these caveats as (Pat del?). (DOCX) [file pgen.1004139.s004.docx]

| **Subject** | **Marker** | **Band** | **Proband** | **Mother** | **Father** | **Inheritance** |
| --- | --- | --- | --- | --- | --- | --- |
|  |  |  |  |  |  |  |
| 18q-34c | D18S539 | 18q21.2 | 253*/264 | 260/264 | 253 | Pat dup |
| 18q-34c | D18S69 | 18q21.31 | 197*/203 | 193/203 | 197/201 | Pat dup |
| 18q-34c | D18S39 | 18q21.31 | 207/219* | 207 | 207/219 | Pat dup |
| 18q-34c | D18S1144 | 18q21.31 | 170*/178 | 168/178 | 166/170 | Pat dup |
| 18q-34c | D18S1129 | 18q21.32 | 251*/256 | 254/256 | 251/253 | Pat dup |
| 18q-34c | D18S477 | 18q22.1 | 241 | 241/245 | 243/249 | Pat del |
| 18q-34c | D18S466 | 18q22.1 | 211 | 209/211 | 209/211 | U |
| 18q-34c | D18S1092 | 18q22.2 | 163 | 157/163 | 163 | U |
| 18q-34c | D18S848 | 18q22.3 | 96 | 96 | 92/96 | U |
| 18q-34c | D18S1091 | 18q22.3 | 242 | 242 | 242 | U |
|  |  |  |  |  |  |  |
| EGL044 | D2S2189 | 2q33.3 | 278 | 278 | 278 | U |
| EGL044 | D2S371 | 2q34 | 146 | 146 | 146 | U |
| EGL044 | D2S334 | 2q34 | 114 | 114/122 | 114/122 | U |
| EGL044 | D2S301 | 2q35 | 222*/232 | 230/232 | 222/232 | Pat dup |
| EGL044 | D2S2204 | 2q36.6 | 232 | 230/232 | 232 | U |
| EGL044 | D2S395 | 2q37.3 | 159 | 159 | 157 | Pat del |
| EGL044 | D2S2285 | 2q37.3 | 251 | 251/252 | 251 | U |
| EGL044 | D2S125 | 2q37.3 | 96 | 93/96 | 89/100 | Pat del |
| EGL044 | D2S140 | 2q37.3 | 180 | 180 | 180 | U |
| EGL044 | D2S2985 | 2q37.3 | 150 | 150/156 | 156 | Pat del |
|  |  |  |  |  |  |  |
| EGL398 | D2S395 | 2q37.3 | 157/162* | 155/157 | 157/162 | Pat dup |
| EGL398 | D2S2285 | 2q37.3 | 260 | 255/260 | 260 | U |
| EGL398 | D2S140 | 2q37.3 | 180 | 180 | 180 | U |
| EGL398 | D2S2985 | 2q37.3 | 152/156* | 149/152 | 152/156 | Pat dup |
| EGL398 | D2S2585 | 2q37.3 | 178 | 178/181 | 177 | Pat del |
| EGL398 | SHGC-74115 | 1p36.33 | 102 | 102 | 102 | U |
| EGL398 | A001Y42 | 1p36.33 | 134/212* | 134/212 | 134/212 | U |
| EGL398 | D1S2515 | 1p36.33 | 102 | 102 | 102 | U |
| EGL398 | D1S243 | 1p36.33 | 136*/161 | 151/161 | 136 | Pat trans |
| EGL398 | D1S468 | 1p36.32 | 177*/187 | 181/187 | 171/177 | Pat trans |
|  |  |  |  |  |  |  |
| 18q-207c | D18S474 | 18q21.2 | 120*/132 | 122/132 | 120/130 | Pat dup |
| 18q-207c | D18S984 | 18q21.2 | 148 | 148 | 148 | U |
| 18q-207c | D18S1110 | 18q21.2 | 274*/288 | 274/288 | 274/286 | Pat dup |
| 18q-207c | D18S1358 | 18q22.3 | 135 | 135 | 139 | Pat del |
| 18q-207c | D18S1106 | 18q22.3 | 173 | 171/173 | 163/171 | Pat del |
| 18q-207c | D18S485 | 18q22.3 | 179 | 179/181 | 179/181 | U |
| 18q-207c | D18S469 | 18q22.3 | 231 | 231 | 237 | Pat del |
| 18q-207c | D18S541 | 18q22.3 | 272 | 268/272 | 272/280 | U |
|  |  |  |  |  |  |  |
| 18q-223c | G15802 | 18q21.33 | 134*/146 | 134/146 | 134/150 | Pat dup |
| 18q-223c | D18S1270 | 18q21.33 | 271*/282 | 271/282 | 271/286 | Pat dup |
| 18q-223c | D18S68 | 18q21.33 | 268*/274 | 268/274 | 268/278 | Pat dup |
| 18q-223c | D18S1358 | 18q22.3 | 139 | 139 | 123/139 | U |
| 18q-223c | D18S1106 | 18q22.3 | 163 | 163/171 | 163/175 | U |
| 18q-223c | D18S874 | 18q22.3 | 183 | 179/183 | 179/183 | U |
| 18q-223c | D18S485 | 18q22.3 | 183 | 183 | 173 | Pat del |
| 18q-223c | D18S469 | 18q22.3 | 233 | 233 | 231/233 | U |
| 18q-223c | D18S848 | 18q22.3 | 95 | 95 | 87/95 | U |
|  |  |  |  |  |  |  |
| 18q-62c | D18S1142 | 18q22.1 | 164/168* | - | 164/168 | U |
| 18q-62c | D18S483 | 18q22.1 | 217 | - | 215/217 | U |
| 18q-62c | D18S1113 | 18q22.1 | 206/214* | - | 212/214 | Pat dup |
| 18q-62c | D18S465 | 18q22.1 | 238/242* | - | 242/244 | Pat dup |
| 18q-62c | D18S969 | 18q22.1 | 356/360* | - | 354/360 | Pat dup |
| 18q-62c | D18S1358 | 18q22.3 | 123 | - | 135/139 | Pat del |
| 18q-62c | D18S1106 | 18q22.3 | 171 | - | 173/175 | Pat del |
| 18q-62c | D18S874 | 18q22.3 | 183 | - | 179/183 | U |
| 18q-62c | D18S485 | 18q22.3 | 181 | - | 177/181 | U |
| 18q-62c | D18S469 | 18q22.3 | 231 | - | 236 | Pat del |
| 18q-62c | D18S541 | 18q22.3 | 272 | - | 268/272 | U |
|  |  |  |  |  |  |  |
| 18q-119c | D18S1357 | 18q21.32 | 121 | 121 | - | U |
| 18q-119c | D18S1148 | 18q21.33 | 143 | 143 | - | U |
| 18q-119c | D18S1134 | 18q21.32 | 209 | 209 | - | U |
| 18q-119c | D18S1147 | 18q21.33 | 204*/213 | 213 | - | Pat dup |
| 18q-119c | D18S1358 | 18q22.3 | 213 | 213 | - | Pat del? |
| 18q-119c | D18S1106 | 18q22.3 | 163 | 163/171 | - | Pat del? |
| 18q-119c | D18S874 | 18q22.3 | 183 | 183 | - | Pat del? |
| 18q-119c | D18S485 | 18q22.3 | 183 | 183 | - | Pat del? |
| 18q-119c | D18S469 | 18q22.3 | 233 | 233 | - | Pat del? |
| 18q-119c | D18S541 | 18q22.3 | 284 | 280/284 | - | Pat del? |
|  |  |  |  |  |  |  |
| 18q-107c | D18S1092 | 18q22.2 | 162 | 157/162 | 156/162 | U |
| 18q-107c | D18S979 | 18q22.1 | 160/164* | 160 | 164 | Pat dup |
| 18q-107c | ATA82B02 | 18q22.3 | 179*/188 | 188 | 179/188 | Pat dup |
| 18q-107c | D18S466 | 18q22.1 | 209 | 209/211 | 209/211 | U |
| 18q-107c | D18S1365 | 18q22.1 | 222/226* | 222/226 | 222/226 | U |
| 18q-107c | D18S1091 | 18q22.3 | 241 | 241 | 241 | U |
| 18q-107c | D18S1358 | 18q22.3 | 123 | 123/139 | 135/139 | Pat del |
| 18q-107c | D18S1106 | 18q22.3 | 163 | 163 | 163/171 | U |
| 18q-107c | D18S874 | 18q22.3 | 175 | 175/183 | 175/183 | U |
| 18q-107c | D18S485 | 18q22.3 | 179 | 179 | 177/179 | U |
| 18q-107c | D18S469 | 18q22.3 | 231 | 231/233 | 233/237 | Pat del |
|  |  |  |  |  |  |  |
| EGL106 | D5S2488 | 5p15.33 | 234 | 231/234 | - | Pat del? |
| EGL106 | D5S392 | 5p15.33 | 93 | 93 | - | Pat del? |
| EGL106 | D5S406 | 5p15.32 | 165 | 165/178 | - | Pat del? |
| EGL106 | D5S2081 | 5p15.2 | 196 | 194/196 | - | Pat del? |
| EGL106 | D5S1991 | 5p15.2 | 225 | 225/228 | - | Pat del? |
| EGL106 | D5S486 | 5p15.1 | 88 | 88 | - | Pat del? |
| EGL106 | D5S411 | 5p14.3 | 158 | 138/158 | - | Pat del? |
| EGL106 | D5S2845 | 5p14.3 | 153 | 153 | - | Pat del? |
| EGL106 | D5S813 | 5p14.2 | 232 | 228/232 | - | Pat del? |
| EGL106 | D5S648 | 5p14.1 | 119 | 119 | - | U |
| EGL106 | D5S814 | 5p14.1 | 183/191* | 183/187 | - | Pat dup |
| EGL106 | D5S821 | 5p14.1 | 168*/177 | 168/177 | - | U |
| EGL106 | D5S1502 | 5p14.1 | 240 | 240 | - | U |
| EGL106 | D5S419 | 5p14.1 | 203*/213 | 213 | - | Pat dup |
| EGL106 | D5S627 | 5p14.1 | 250*/256 | 254/256 | - | Pat dup |
| EGL106 | D5S2113 | 5p14.1 | 254*/256 | 256 | - | Pat dup |
| EGL106 | D5S1758 | 5p14.1 | 225 | 225 | - | U |
